# Pseudoaneurysm of the mitral–aortic intervalvular fibrosa presenting after chest trauma and diagnosed by cardiac magnetic resonance: a case report

**DOI:** 10.1186/1752-1947-6-357

**Published:** 2012-10-19

**Authors:** Adriana Dias Barranhas, Márcia Cláudia Dias, Alair Augusto Sarmet Moreira Damas dos Santos, Edson Marchiori, Marcelo Souto Nacif

**Affiliations:** 1 Radiology Department, Federal Fluminense University School of Medicine, HUAP 2º andar, Rua Marquês do Paraná s/n., Centro - Niterói, RJ, Brazil; 2 ProEcho Cardiodata Serviços Médicos, Unidade Niterói, Rua Jornalista Moacir Padilha 195, Centro - Niterói, RJ, Brazil; 3 Radiology Department, Rio de Janeiro Federal University, Rua Professor Rodolpho Paulo Rocco, 255. – Ilha do Fundão, Rio de Janeiro, RJ, Brazil; 4 Radiology and Imaging Sciences, National Institutes of Health Clinical Center, 10 Center Drive, Building 10, BIN264B, Bethesda, MD, USA; 5 Division of Cardiology, Johns Hopkins University School of Medicine, 600 N. Wolfe Street, Blalock, Baltimore, MD, USA

**Keywords:** Heart defects, Congenital, Magnetic resonance imaging, Aneurysm

## Abstract

**Introduction:**

Annular subvalvular pseudoaneurysm is a rare example of left ventricle aneurysm described predominantly in young African people. These aneurysms are divided into two different types, namely, submitral or subaortic, with subaortic being the less frequent kind. The subaortic type is most often localized in the mitral–aortic intervalvular fibrosa. To the best of our knowledge, this is the first report of a mitral–aortic intervalvular fibrosa pseudoaneurysm associated with coarctation of the aorta, anomalous pulmonary venous return, bicuspid aortic valve and patent ductus arteriosus diagnosed by cardiovascular magnetic resonance.

**Case presentation:**

We report the case of a 15-year-old African-American boy with a history of mild chest trauma who underwent echocardiographic evaluation as part of an out-patient work up. The echocardiogram was suspicious for the presence of mitral-aortic intervalvular fibrosa pseudoaneurysm and cardiovascular magnetic resonance was then performed to better characterize this finding. In addition to confirming the presence of the aneurysm, cardiovascular magnetic resonance also revealed coarctation of the aorta, a bicuspid aortic valve, and anomalous pulmonary venous return.

**Conclusion:**

In our case, cardiovascular magnetic resonance was helpful in: (a) making a definite diagnosis of mitral–aortic intervalvular fibrosa pseudoaneurysm and its borders, which was not clear with an echocardiogram examination; and (b) illustrating additional associated congenital anomalies including the anomalous pulmonary venous return.

## Introduction

Subvalvular aneurysm (SA), first reported in 1813, is a rare type of left ventricle aneurysm and is classified into submitral and subaortic [1,2]. The subaortic type may protrude into the left atrium [3] and/or left ventricular outflow tract (LVOT) [4].

A transthoracic echocardiogram (TTE) is the most validated method to assess SA although SA is also discernible with other imaging modalities such as transesophageal echocardiography (TEE) [3-5]. Cardiovascular magnetic resonance (CMR), a non-invasive and highly reproducible modality, has been increasingly available in clinical cardiology [6,7]. However, few studies involving CMR and SA are available in peer-reviewed publications. Currently, there are slightly more than 100 published cases of which less than 30% are of the subaortic type, and the vast majority is related to complicated inflammatory processes or post-surgery complications [4,6,8].

In this study we report a case of an African-American young patient with a history of mild chest trauma in out-patient follow-up who had the diagnosis of subvalvular aortic pseudoaneurysm, in the mitral–aortic intervalvular fibrosa (MAIVF), associated with coarctation of the aorta, bicuspid aortic valve and anomalous pulmonary venous return by CMR.

## Case presentation

**Figure 1:**
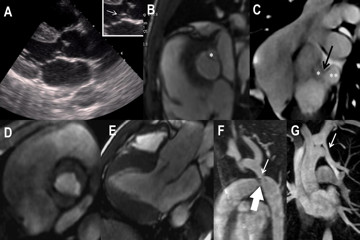
**Imaging findings of subvalvular aneurysm and associated congenital defects.** (**A**) Echocardiogram in the plane of left ventricular outflow tract (LVOT) observed (highlighted) an aneurysm formation in the topography of subvalvular mitral–aortic fibrosa (arrow). (**B**, **C**, **D** and **E**) Cine-cardiovascular magnetic resonance to study the region of mitral–aortic intervalvular fibrosa. (**B**) Subaortic plane showing inner portion (*) of subvalvular pseudoaneurysm. (**C**) Coronal oblique plane, specific to the topography of the lesion, where the inner portion (*), neck (black arrow) and intrapericardial portion (**) of the pseudoaneurysm can be observed. (**D**) Valve plane during systole showing the opened bicuspid aortic valve. (**E**) LVOT plane individualizing mild aortic regurgitation, this plane does not have the same angle of echocardiography and therefore does not demonstrate the lesion. (**F** and **G**) Magnetic resonance angiography of the thorax. (**F**) Patent arteriosus ductus (large arrow) and coarctation of the aorta (arrow). (**G**) Anomalous pulmonary venous return of the left upper lobe vein into the innominate artery on the same side (arrow).

A 15-year-old asymptomatic African-American boy was referred to our Emergency room after chest trauma during a football match. A physical examination revealed a heart murmur prompting further cardiovascular testing. A chest radiograph was normal. A subsequent two-dimension TEE showed an echogenic image with defined edges adjacent to LVOT in the region of the mitral–aortic junction with no LVOT gradient and/or shunts, suggesting pseudoaneurysm of the MAIVF associated with bicuspid aortic valve (Figure 1). The patient was then sent for further study with CMR.

The CMR was performed in a GE Healthcare 1.5 Tesla (T) Signa HDxT EchoSpeed Plus R (General Electric, Milwaukee, USA) with gradient of high performance using 32mT of amplitude and with 150T/m/s of variation. The entire study was triggered with electrocardiogram and expiratory apnea. Cine-magnetic resonance (MR) to study the function was performed with the basic planes [7] and focused on the region of interest through the steady-state free precession sequences using the following technical parameters: repetition time (TR) 3.1ms, 1.55ms echo time (TE), flip angle 55°, field of view 350 to 420mm, matrix 192 × 128, number of cardiac phases 20, number of excitations (NEX) 1, number of slices 10, slice thickness 8mm, and the interval between slices (gap) 2mm. This scan identified the TEE finding as a subvalvular subaortic pseudoaneurysm located at MAIVF.

Furthermore, CMR evidenced a protrusion to the pericardial cavity, presenting neck of 8mm, depth of 10mm and width of 15mm (Figures 1B and 1C). The aortic valve was found to be bicuspid with mild regurgitation (Figures 1D and 1E). The patient also underwent MR angiography using the three-dimensional gradient echo technique with intravenous contrast and the following parameters: thickness of 2.4mm, 192 × 256mm matrix, echo time 1.0ms, repetition time 4.6ms, flip angle 45°, and NEX 1. The paramagnetic contrast used was gadoversetamide (Gd-DTPA-BMEA; Mallinckrodt Inc., USA) with a dose of 0.2mmol/kg and infusion velocity of 2.5ml/s. The sequence was repeated three times at intervals of 30 seconds between each breath hold in order to assure precocious and late acquisitions.

This technique identified the presence of a pre-ductal coarctation of approximately 1.9cm from the left subclavian artery origin with minimum caliber of 0.7cm. The precoarctation caliber was 1.3cm and the postcoarctation caliber was 2.6cm. A small patent ductus arteriosus, as well as an extensive network of collaterals, could also be identified (Figure 1F). The patient possessed two pulmonary veins in the right and one pulmonary vein in the left draining into the left atrium. Another finding by MR angiography was partial anomalous venous return from the left upper lobe to the left brachiocephalic vein (Figure 1G). The whole examination took 50 minutes and there were no complications.

The patient was diagnosed with MAIVF pseudoaneurysm associated with aortic coarctation, bicuspid aortic valve, patent ductus arteriosus and anomalous pulmonary venous return. In addition, he was referred to the cardiothoracic surgery department where it was decided to follow the patient with repeat imaging because there were no acute surgical indications. A 6-months clinical and imaging evaluation follow-up has already been performed without changes.

## Discussion

SA is extremely rare and hence the importance of new case reports. With the advent of new diagnostic tests, such as CMR, an early diagnosis could avoid cases such as the one reported by Corvisart [2] in which necropsy revealed an aneurysm "almost the size of the heart."

In theory, SA by pathology can be divided into true [9] and false [10] aneurysms differentiated by the formed layers. In addition, it seems that true aneurysms are mostly associated with congenital cases [1,8], whereas false aneurysms, also called pseudoaneurysms, are more commonly associated with post-surgery and/or trauma and post-infectious complications [3,4,11]. However, there is a lot of discussion about this and some authors still use the term aneurysm to describe pseudoaneurysms [12]. In reality, identifying the formed layers is extremely difficult using either imaging modality (echocardiography or magnetic resonance imaging), and the general description of SA may still be used [13].

The MAIVF is one of the possible regions to observe SA. The MAIVF is a fibrous region of the heart with great clinical and surgical importance because it is located between the anterior leaflet of the mitral valve and the non-coronary and left coronary cusps. The MAIVF is therefore correlated with the anatomical and functional integrity of both valves [9].

Complications of the aneurysm and/or pseudoaneurysm exist, such as perforation with shunt of LVOT into the left atrium, infection, compression of the coronary or pulmonary arteries; also, rapid increase of size with the possibility of rupture, embolization, or primary valvular dysfunctions are indications for surgery in these patients [8].

Abrahams *et al*. [1] and Chesler *et al*. [9,10] provided better understanding of clinical and pathophysiological aspects of these aneurysms. SA was erroneously defined as a disease of young Black people with congenital etiology, probably with weakness of the ventricular wall in the atrioventricular groove. Nowadays, it is known that despite the higher prevalence in Blacks, cases have also been reported in Whites and even in Brazilian Indians [4,5].

**Figure 2:**
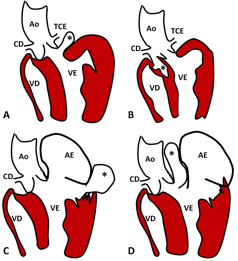
Representation of possible locations of the subvalvular ventricular aneurysm (*). (**A**) Subaortic, below the aortic annulus. Another presentation can be supra-aortic, in the left aortic sinus. Both near the left coronary artery. (**B**) Supra-aortic, perforation in the right aortic sinus insinuating itself into the interventricular septum (IVS). In general, when these defects are subaortic, they are complete and result in a defect of IVS. (**C**) Congenital alterations in fibromuscular junction of the mitral ring resulting in submitral aneurysm. (**D**) Perforation in fibrosa union between the base of the anterior mitral leaflet and the aortic root resulting in an aneurysm between the aorta and left atrium. This last example represents our case report, but we had an insinuation into the left ventricular outflow tract. Ao = aorta, AE = left atrium, CD = right coronary artery, TCE = left coronary artery; VD = right ventricle, VE = left ventricle.

The SA is a rare example of a left ventricular aneurysm with a submitral or subaortic location (Figure 2), in which its etiology is poorly defined but unrelated to coronary artery disease. Some causes are proposed [1,9,10]: muscle weakness in congenital atrioventricular groove, anomalous origin of coronary arteries, trauma, polyarteritis nodosa, rheumatic carditis, infectious endocarditis, tuberculosis and syphilis.

In chest radiographs a bulge in the left cardiac silhouette of varying size and shape can be observed according to the size and position of the aneurysm [1]. Currently, echocardiography is the method of choice for the evaluation of SA [9,11]. However, when doubtful or difficult cases occur, the study with CMR is a more beneficial method for evaluation.

In this case we described an association of several findings as MAIVF pseudoaneurysm, aorta coarctation, anomalous pulmonary venous return, bicuspid aortic valve and patent ductus arteriosus. The association of all findings strongly suggests a congenital syndrome. For example, an incomplete Shone’s syndrome could explain the major associations [14,15]. In addition, in this case, our hypothesis about the MAIVF pseudoaneurysm formation is that chest trauma in the context of congenital fragility increases the likelihood of pseudoaneurysm formation. The choice for a close follow-up and re-evaluation for a possible surgery procedure in our patient is in accordance with 9% of all pseudoaneurysms of MAIVF cases reported in the literature. A long-term follow-up showed that surgical intervention is not mandatory in asymptomatic patients [11]. However, to date the most common treatment (63%) is surgery with repair of the MAIVF [4].

## Conclusion

In our case, CMR along with MR angiography were helpful in: (a) making a definite diagnosis of MAIVF and its borders, which was not clear with a two-dimension TTE examination; (b) identifying additional associated congenital anomalies; and (c) visualizing the anomalous pulmonary venous return that was not previously detected. In this case, the multimodality imaging approach significantly improved our evaluation of the patient. To the best of our knowledge, this is the first report of a MAIVF aneurysm associated with aorta coarctation, anomalous pulmonary venous return, bicuspid aortic valve and patent ductus arteriosus diagnosed by CMR.

## Consent

Written informed consent was obtained from the patient’s legal guardian for publication of this case report and accompanying images. A copy of the written consent is available for review by the Editor-in-Chief of this journal.

## Competing interests

The authors declare that they have no competing interests.

## Authors’ contributions

ADB: study design, CMR acquisition, CMR analysis, CMR interpretation, and manuscript drafting; MCD: echocardiography acquisition, echocardiography analysis, and manuscript revision; AASMDS: CMR interpretation and manuscript revision; EM: CMR interpretation and manuscript revision; MSN: principal investigator, study design, CMR acquisition, CMR interpretation and manuscript revision. All authors read and approved the final manuscript.
